# Role of curcumin in ischemia and reperfusion injury

**DOI:** 10.3389/fphar.2023.1057144

**Published:** 2023-03-20

**Authors:** Minglei Bi, Danyi Li, Jin Zhang

**Affiliations:** ^1^ Department of Plastic Surgery, Lanzhou University Second Hospital, Lanzhou, Gansu, China; ^2^ Department of Ophthalmology, Jiading District Central Hospital, Shanghai University of Medicine and Health Sciences, Shanghai, China

**Keywords:** curcumin, ischemia-reperfusion injury, oxidative stress, inflammation, cell death

## Abstract

Ischemia-reperfusion injury (IRI) is an inevitable pathological process after organic transplantations. Although traditional treatments restore the blood supply of ischemic organs, the damage caused by IRI is always ignored. Therefore, the ideal and effective therapeutic strategy to mitigate IRI is warrented. Curcumin is a type of polyphenols, processing such properties as anti-oxidative stress, anti-inflammation and anti-apoptosis. However, although many researches have been confirmed that curcumin can exert great effects on the mitigation of IRI, there are still some controversies about its underlying mechanisms among these researches. Thus, this review is to summarize the protective role of curcumin against IRI as well as the controversies of current researches, so as to clarify its underlying mechanisms clearly and provide clinicians a novel idea of the therapy for IRI.

## 1 Introduction

IRI is one of the most important clincial issues that limits the development of organic transplantations and tissue repair. Although it is critical to restore the blood supply of ischemic tissuses promptly, therapies targeting on the exact mechanisms of IRI-induced damage could make further improvements on the clinical efficacy.

During ischemia phase, intracellular mitochondrial oxidative phosphorylation induces the anaerobic metabolism, acidification and ion channels dysfunctions (Sarighieh et al., 2020). Subsequently, intracellular Ca^2+^ overload and abnormal hyperosmosis both occur and thus lead to the cell injury (Wu et al., 2018). During reperfusion phase, the excessive activation of oxidative stress and inflammation as well as Ca^2+^ overload seriously impair the structural integrity of outer membranes of mitochondia, which leads to the opening of the mitochondrial permeability transition pore (mPTP), resulting in the release of cytochrome C into cytoplasm and thus triggers apoptosis (Bonora et al., 2022; Fedotcheva et al., 2022). Simultaneously, the excessive free radicals leads to the lipid peroxidation of membranous structure, impairment of the structures and functions of proteins and nucleic acid, causing cell injury (Daverey et al., 2020).

**FIGURE 1:**
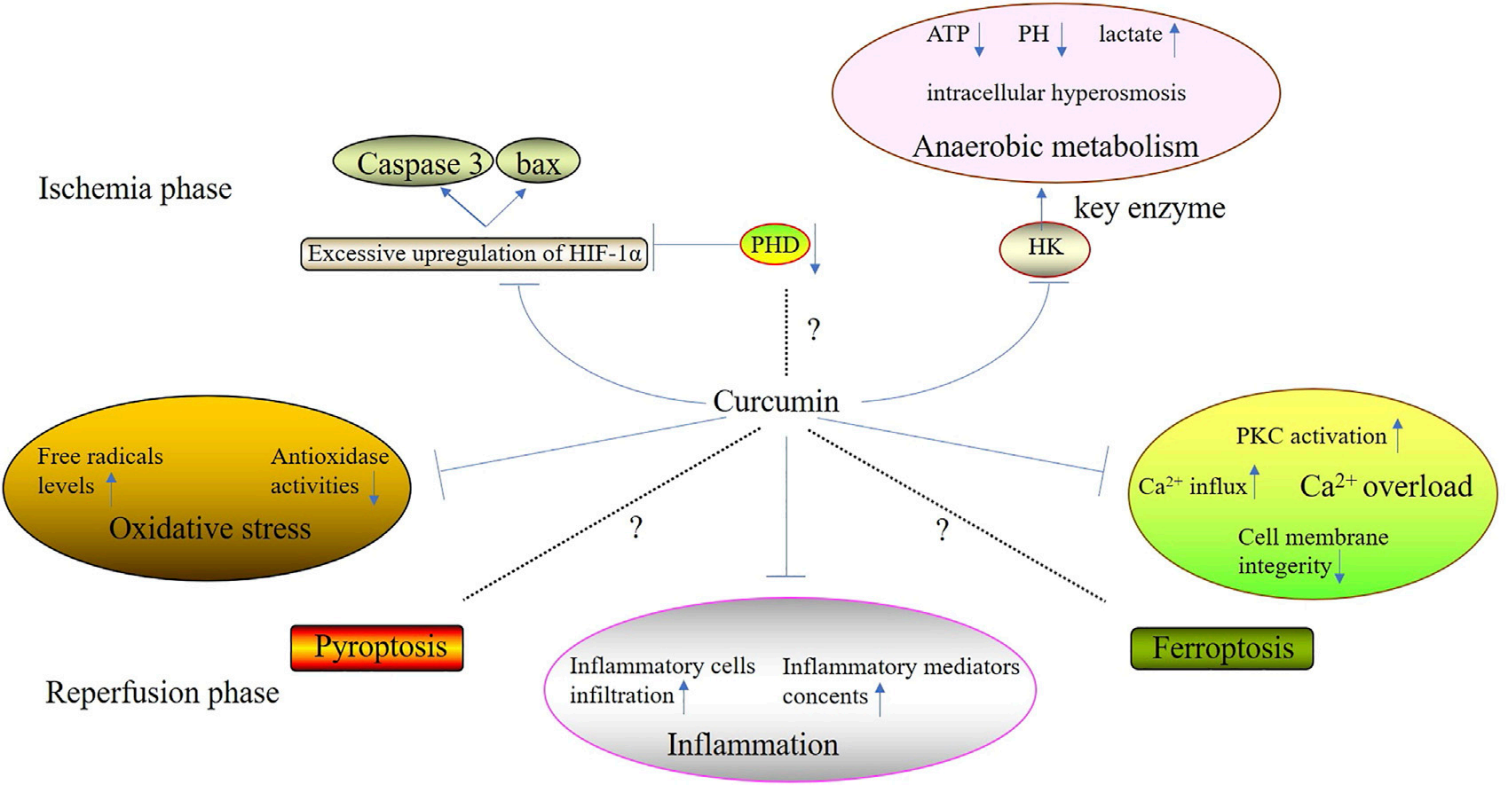
The effectiveness therapeutiveness of curcumin to attenuate organic IRI.

Curcumin is isolated from turmeric and its molecular formula is C_21_H_20_O_6_, of which the main chain is unsaturated fat and aromatic family groups (Daverey et al., 2020). Compared with other types of Traditional Chinese Medicine, curcumin has a huge advantage because it processes several biological properties such as scavenging free radicals, anti-inflammation, anti-apoptosis, etc., (Pang et al., 2022). Recently, it has been accepted that curcumin can alleviate cell injury during ischemic phase and suppress the excessive oxidative stress and inflammation during reperfusion phase (Figure 1) (Table 1). However, there is still some controversies over the mechanisms of the suppression of curcumin on cell death.

**TABLE 1:** Characteristics of studies included in this review.

Animal models/Cell models/Experimental techniques	Drug administrations	Dose/Concentration	Mechanisms	References
Cell injury				
Chronic intermittent hypoxia-induced myocardial injury mice model	Oral gavage	100 mg/kg	Preventing myocardial cell death signalling	Moulin et al. (2020)
Hypoxia/reoxygenation (H/R) treatment in rat bone marrow mesenchymal stem cells	Mixed with medium	10 mM	Preventing H/R injury by protecting mitochondrial function, destabilization of HIF-1α and activation of Epac1-Akt signaling pathway	Wang et al. (2019)
Human colorectal cancer cells	Mixed with medium	10, 20, 40 and 50 μmol/L	Inhibiting aerobic glycolysis and inducing mitochondrial-mediated apoptosis through hexokinase II	Wang K. et al. (2015)
N-Methyl N-Nitrosourea induced neurotoxicity on mouse cerebellum and cerebrum	Oral gavage	60 mg/kg	Reducing the activities of carbohydrate metabolizing enzymes	Singla et al. (2010)
Oxidative stress				
Hypoxia/reoxygenation treatment in H9C2 cells	Mixed with medium	5, 10 and 20 μM	Downregulating Notch signaling	Zhu et al. (2019)
Renal ischemia and reperfusion injury rat model	Oral gavage	200 mg/kg	Antioxidant effects	Bayrak et al. (2008)
Cerebral ischemia/reperfusion injury rat model	Intraperitoneal injection	10, 20 and 40 mg/kg	Attenuating oxidative stress, inflammation, and apoptosis and activating endogenous antioxidant defenses	Wicha et al. (2017)
TGF-β1 mediated basal alveolar epithelial cells epithelial mesenchymal transition	Mixed with medium	20 μM	Triggering the p53-fibrinolytic system	Shaikh et al. (2021)
Hepatocellular carcinoma cells	Mixed with medium	10, 20 and 40 μM	Regulating the TET1/Wnt/β-catenin signal pathway	Zhu et al. (2019)
Middle cerebral artery occlusion (MCAO) rat model	Intraperitoneal injection	100 mg/kg	The neurogenesis-related lncRNA/circRNA-miRNA-mRNA ceRNA networks	Li et al. (2022)
Isolated guinea pig hearts ischemia and reperfusion injury	Mixed with perfusion buffer	0.2 and 0.5 𝜇M	Attenuating activities of GPx and GR	Ilyas et al. (2016)
Liver warm ischemia and reperfusion injury rat model	Injecting into a branch of superior mesenteric vein	50 mg/kg	Regulation of heat shock protein and antioxidant enzymes	Shen et al. (2007)
Computational method	--	--	Increasing catalase activity of bovine liver catalase	Mofidi et al. (2017)
Multispectral analysis and simultaneous docking simulations	--	--	Inhibiting catalase activity	Khataee et al. (2019)
Inflammation				
Liver damage rat model	Oral gavage	30 mg/kg	Suppressing the levels of AST, LDH, HDL, LDL, triglyceride, and total cholesterol in serum, and fibrosis, caspase-3, Bax, and TNF-α expressions in the liver	Huyut et al. (2022)
Renal ischemia-reperfusion injury rat model	Oral gavage	--	Regulating expression level of macrophage subtypes	Fan et al. (2019)
Human umbilical vein endothelial cell exposed to urban particulate material or titanium dioxide nanoparticles	Mixed with medium	1, 10 and 100 μM	Suppressing the expression of E− and P-selectins, ICAM-1, VCAM-1 and platelet-endothelial cell adhesion molecule-1	Montiel-Dávalos et al. (2017)
Ischemia and reperfusion injury rabbit ear wounds	Intravenously administration	6, 30 and 60 μg/kg	Decreasing proinflammatory cytokines IL-1, IL-6 and IL-8	Jia et al. (2014)
Ischemia and reperfusion injury retina model	Diet	100, 500 and 2,500 ppm in diets	Inhibiting injury-induced activation of NF-kB and STAT3, and on over-expression of MCP-1	Wang et al. (2011)
Myocardial ischemia and reperfusion injury rat model	Oral gavage	300 mg/kg	Inhibiting the expression of TLR2	Kim et al. (2012)
Human placenta, visceral adipose tissue (VAT) and subcutaneous adipose tissue (SAT) explants	Mixed with medium	60 μM	Supressing proinflammatory cytokines (IL1A, IL1B, and IL6) and chemokine (CCL2-4, CXCL1, CXCL5 and CXCL8) and increased anti-inflammatory cytokine IL4 and IL13	Nguyen-Ngo et al. (2020)
Limb ischemia and reperfusion injury rat model	Intraperitoneal injection	200 mg/kg	Regulating miR-21/TLR4/NF-κB signalling pathway	Zou et al. (2020)
Atherosclerotic lesions at the aortic sinus mice model	Diet	0.1% diet	Inhibiting Toll-like receptor 4 expression	Zhang et al. (2018)
Endotoxic mice model	Intravenous injection	10 mg/kg	Blocking the LPS-TLR4/MD2 signaling activation	Wang Y. et al. (2015)
Histamine-induced itching mice model	Topical application	10 mg/mL	Activation of transient receptor potential vanilloid 1	Lee et al. (2018)
Rat basophil leukemia (RBL)-2H3 and human pre-basophils (KU812) cell lines	Mixed with medium	0.2–200 μg/mL	Inhibiting FceRI protein expression and protein kinase C delta translocation	Kong D. et al. (2020)
Pentylenetetrazol-induced seizures	Intraperitoneal injection	150 mg/kg	Blocking 5-HT1A, 5-HT2C, 5-HT4 and 5-HT7 receptors	Arbabi et al. (2018)
Cardiotoxicity rat model	Oral gavage	50 mg/kg	Antioxidant effect	Mohammed et al. (2020)
Molecular modeling and docking techniques	--	--	Inhibiting phospholipase A2	Dileep et al. (2011)
Review	--	--	Inhibiting IL-1, IL-2, IL-6, IL-8 and IL-12, TNF-α, monocyte chemoattractant protein-1	Kocaadam et al. (2017)
Cerebral ischemia and reperfusion injury rat model	Intraperitoneal injection	300 mg/kg	Inhibiting inflammation and apoptosis	Li et al. (2017)
Intestinal ischemia and reperfusion injury rat model	left femoral vein administration of	1 and 5 mg/kg	Inhibiting NF-𝜅B signaling pathway	Fan et al. (2014)
Mesenteric ischemia and reperfusion injury rodent model	Oral gavage	40 mg/kg	Suppressing oxidative stress and increased HSP 70	Karatepe et al. (2009)
Cerebral ischemia and reperfusion injury rat model	Oral gavage	100 and 300 mg/kg	Downregulation of miR-7-5p	Xu et al. (2019)
Liver ischemia and reperfusion injury mice model	Intraperitoneal injection	100 mg/kg	Activation of peroxisome proliferator-activated receptor	Liu et al. (2018)
Limb ischemia and reperfusion rat model	Femoral blood injection	200 mg/kg	Suppressing Notch2/Hes-1 signaling pathway	Bo et al. (2020)
Ca^2+^ overload				
MCAO rat model	Oral gavage	100 mg/kg	Suppressing the activation of P2X7 receptor	Wang X. et al. (2020)
Albumin-induced cell stress and proinflammatory response	Mixed with medium	10 μM	Inhibiting Ca^2+^ overload	Nazıroğlu et al. (2019)
H_2_O_2_-induced oxidative dress in astrocytes	Mixed with medium	10 μM	Inhibiting Ca^2+^ influx	Daverey et al. (2020)
Human breast carcinoma cells	Mixed with medium	1 mg/mL	Inhibiting PI3k/AKT/mTOR and RhoA/ROCK signaling pathways	Badr et al. (2018)
Mouse fibroblast cells	Mixed with medium	15 and 20 μM	Suppressing PKC activity	Liu et al. (1993)
Cochlear fibroblasts of diabetic rat model	Oral gavage	200 and 400 mg/kg	Inhibiting PKC expression	Haryuna et al. (2019)
Human pulmonary artery smooth muscle cells	Mixed with medium	20 μM	Inhibiting PLD activity	Chakraborti et al. (2018)
Neonatal hypoxic-ischemic brain injury mice model	Intraperitoneal injection	22, 44, 100, 200 and 400 ug/ul	Inhibiting inflammation and oxidative stress	Rocha-Ferreira et al. (2019)
Autophagy				
OGD/R-treated primary cortical neurons	Mixed with medium	2, 5, 10 and 20 μM	Up-regulating AKT/mTOR signalling pathway and decrease autophagy	Shi et al. (2019)
OGD/R-treated cardiomyocytes	Mixed with medium	0.1, 1 and 10 μM	Down-regulating AMPK/mTOR signaling pathway and Promoting Autophagy	Yang et al. (2013)
MCAO rat model	Intraperitoneal injection	20 mg/kg	Promotion of P62-LC3-Autophagy	Zhang et al. (2019)
MCAO rat model	Intraperitoneal injection	100 mg/kg	Regulating Mitophagy and Preserving Mitochondrial Function	Wang Z. et al. (2020)
Hepatic fibrosis rat model	Intraperitoneal injection	100, 200 and 400 mg/kg	Up-regulating signaling pathways of autophagy AMPK and PI3K/AKT/mTOR	Kong Z. L. et al. (2020)
Pyroptosis				
Rat necrotising enterocolitis model	Oral gavage	20 and 50 mg/kg	Activating SIRT1/NRF2 and inhibiting the TLR4 signalling pathway	Yin et al. (2020)
Doxorubicin-induced cardiac injury mice model	Intraperitoneal injection	100 and 200 mg/kg	Activating Akt/mTOR signalling pathway	Yu et al. (2020)
Ferroptosis				
MCAO rat model	Intraperitoneal injection	5 mg/kg	Inhibiting ferroptosis	Tuo et al. (2017)
Doxorubicin-induced cardiac injury mice model	Intraperitoneal injection	1 μM	Upregulation of Hmox1 *via* Nrf2 activation	Fang et al. (2019)
Renal ischemia and reperfusion injury mice model	--	--	Inhibiting ferroptosis	Linkermann (2016)
OGD/R-treated TM4 mouse Sertoli cells	Gene knockdown	--	Activating GPX4 and inactivating p38 MAPK	Li et al. (2018)
Lewis lung carcinoma mice model	Intraperitoneal injection	100 mg/kg	Inhibiting autophagy and ferroptosis	Tang et al. (2021)

## 2 Therapeutic approaches for IRI

Current therapeutic approaches are various and its foundations are alleviating ischemia and restoring reperfusion (Mohamadian et al., 2022). Therapeutic approaches can be divided into surgery, physiotherapy and pharmacology.

With the development of microsurgical techniques, surgery has promoted the prognosis of IRI. Removing thrombus, establishing collateral circulation and anastomosing broken blood vessels are important in alleviating ischemia and restoring reperfusion (Ferreira et al., 2022). For example, percutaneous coronary intervention (PCI) is applied to treat myocardial IRI. And percutaneous transluminal angioplasty (PTA), endarterectomy and bypass operation are applied for the treatments of organic IRI caused by vascular diseases.

Mild hypothermia and hyperbaric oxygen are important physiotherapeutic methods for IRI. The main functions of mild hypothermia include decreasing the metabolism and oxygen consumption of organs, alleviating vasopermeability and microcirculation. In clinics, mild hypothermia has been used for the treatments of myocardial IRI and cerebral IRI (Micó-Carnero et al., 2022).

Hyperbaric oxygen therapy refers to the inhalation of pure oxygen under more than one atmosphere, which can increase the blood oxygen concentration and oxygen reserve of tissues, promote the self-update and viability of cells (Neheman et al., 2020). For instance, hyperbaric oxygen therapy is a necessary method for cerebral IRI and chronic wounds.

Types of pharmacological therapies for IRI are various and can be divided into antioxidant and anti-inflammatory agents (Gao et al., 2022). Antioxidant agents can fight against oxidative stress by eliminating free radicals or consuming materials which can produce free radicals, which can be divided into composite and endogenous types. For example, composite antioxidative agents include edaravone, metformin, quercetin, probucol and α-thioctic acid et al. Endogenous antioxidative agents include melatonin, vitamin B, vitamin C, vitamin E and superoxide dismutases et al. Anti-inflammatory agents mainly include leucocyte inhibitors, interleukin-1 (IL-1) inhibitors (prednison, prednisolone and dexamethasone), TNF-α inhibitors, ICAM-1 inhibitors and cyclooxygenase 2 (COX2) inhibitors (Non-steroidal Anti-inflammatory Drugs, NSAIDs).

Surgery has two main disadvantages: expensive and traumatic, so that it is not suitable for elderly patients. Physiotherapy needs professional medical equipments and it usually acts affiliated role in treating IRI. Although pharmacological therapies are various, combinating different mechanisms drugs can exert the best therapeutic effectiveness and some of these durgs have serious side effects (Mohamadian et al., 2022). However, as a classic and popular type of TCM, curcumin has gained a great attention for its multiple mechanisms such as antioxidant, anti-inflammatory, suppressing Ca^2+^ overload and mediating autophagy et al. Therefore, due to its distinctive and various pharmacological properties, curcumin might be the promosing candidate for the treatment of IRI.

## 3 Curcumin attenuates cell injury during ischemia phase

Ischemia phase, the first stage of IRI and ischemia, not only poses direct damage on normal cells, but also lays the foundation for the pathological changes induced by reperfusion injury. During ischemia phase, both the excessive upregulation of hypoxia-inducible factor-1α (HIF-1α) and anaerobic metabolism play significant roles in direct cell injury posed by ischemia. However, multitudes of researches have demonstrated that curcumin can attenuate ischemia-induced cell injury through mediating the mechanisms mentioned above.

### 3.1 Curcumin suppresses the excessive upregulation of HIF-1α

Curcumin is able to suppress the excessive upregulation of HIF-1α, mitigating cell injury caused by ischemia. The foremost change of ischemia is hypoxia which can stimulate the stable expression of HIF-1α, leading to the upregulation and nuclear translocation of HIF-1α (Ding et al., 2022). Subsequently, in nucleoli, HIF-1α combines with hypoxia response elements (HRE) located in the promoter region of hypoxia response genes (HRG), which promotes vascular endothelial growth factor (VEGF) and erythropoietin (EPO) expressions, enhancing the ischemia tolerance (Zhao et al., 2020). However, excessive upregulation of HIF-1α contributes to the expressions of several pro-apoptosis genes such as cysteinyl aspartate specific proteinase 3 (Caspase-3) and BCL2-Associated X Protein (Bax), resulting in apoptosis induced by hypoxia and ischemia (Wang X. et al., 2020). Furthermore, several researches had confirmed that curcumin could mitigate IRI through suppressing the excessive upregulation of HIF-1α. Moulin et al. (2020) found that curcumin attenuated myocardial IRI through suppressing IRI-induced HIF-1 activation in mice. Wang et al. (2019) found that curcumin pretreatment (10 μM) could increase the survival rate of bone derived stromal cells (BMSCs) under IRI condition partly through the induction of HIF-1α destabilization.

In addition, a train of researches has manisfested that during ischemia phase, the suppression of prolyl hydroxylase (PHD) can contribute to the reduction of cellular oxygen consumption and the promotion of cellular ischemia tolerance. Huan et al. (2020) confirmed that the knockdown of PHD1 gene could promote the tolerance of skeletal muscle in mice against ischemia and hypoxia. However, researches investigating curcumin and PHD suppression during ischemia phase are still blank. Therefore, fulfilling this gap is helpful for the comprehensive elucidation of the machanisms of curcumin alleviating IRI.

### 3.2 Curcumin suppresses the anaerobic metabolism

Oxygen deficiency induces the anaerobic metabolism which causes the perturbation of intracellular homeostasis, leading to cell destruction and death (Nguyen et al., 2022). When anaerobic metabolism occurs, there are some corresponding changes in cells including decreasing levels of ATP and PH, increasing lactate concentration and ion channels dysfunctions, resulting in intracellular abnormal acidification and hyperosmosis (Versluis et al., 2022). A series of researches had confirmed that curcumin could suppress anaerobic metabolism, maintaining intracellular homeostasis. Wang K. et al. (2015) showed that curcumin could block anaerobic metabolism and induce mitochondrial-mediated apoptosis in two different lines of human colorectal cancer cells (HCT116 and HT29 cells) in a concentration-dependent manner through the downregulation of the activity of hexokinaseⅡ(HK Ⅱ), a key enzyme of anaerobic metabolism. *In vivo*, (Singla et al., 2010) also showed that curcumin co-administered with *N*-Methyl *N*-Nitrosourea (MNU, one of the potent neuro-carcinogens) could significantly reduce the activity of HK both in the cerebrum and cerebellum of mice.

## 4 Curcumin suppresses the excessive oxidative stress

Curcumin can suppress the excessive oxidative stress induced by IRI effectively. High free radicals levels and low antioxidase activities are characteristic of oxidative stress (Jelic et al., 2021). And its key mechanisms include mitochondrial injury, the aggregation and activation of a huge amount of neutrophils, increase in xanthine oxidase production as well as catecholamin oxidation (Lai et al., 2022). Curcumin can suppress IRI-induced oxidative stress by reducing free radicals levels and enhancing antioxidase activities during reperfusion phase.

### 4.1 Curcumin reduces free radicals levels

Curcumin is a type of effective anti-inflammation agents. The unstable chemical property of free radicals can lead to the lipid peroxidation and destruction of the structures and functions of proteins as well nucleic acid, aggravating organic IRI (Peker et al., 2019). Reactive Oxygen Species (ROS) is the main component of free radicals, including superoxide anion (O^−2^), nitric oxide (NO), hydrogen peroxide (H_2_O_2_), etc. *In vitro*, (Zhu et al., 2019) demonstrated that curcumin could protected H9C2 cardiomyocytes against IRI, reversing the IRI-induced increases in ROS and malondialdehyde (MDA) levels. Bayrak et al. (2008) demonstrated that the oral adminstration of curcumin greatly alleviated renal IRI of rats *via* its inhibition of MDA and NO levels. *In vivo*, (Wicha et al., 2017) demonstrated that treatment with hexahydrocurcumin offered its neuroprotection against IRI in rats through significantly decreasing the levels of MDA and NO of damaged brain tissues. Otherwise, *in vivo*, a series of researches had confirmed that curcumin and its demethoxy derivatives such as demethoxycurcumin (Dmc), bisdemethoxycurcumin (Bdmc), tetrahydrocurcumin (THC), hexahydrocurcumin (HHC) and octahydrocurcumin (OHC) could eliminate 1,1-diphenyl-2-trinitrophenylhydrazine (DPPH), NO, hydroxy radical (OH·) and O^−2^ directly by providing electrons to reduce free radicals (Shaikh et al., 2021; Zhu et al., 2022; Shahbazizadeh et al., 2021). Taken together, curcumin can reduce free radical levels so as to mitigate IRI.

### 4.2 Curcumin enhances antioxidase activities

Curcumin can change the structures of many types of antioxidase such assuperoxide dismutases (SOD), catalase, glutathione reductase (GR) and glutathione peroxidase (GPx) and enhance its expressions and activities. On one hand, (Li et al., 2022). found that chronic curcumin treatment on cerebral IRI in rats could reduce neurological scores and inhibit IRI-induced apoptosis by enhancing enzyme activities of SOD, catalase and GR. Ilyas et al. (2016) also found that curcumin exerted its protective role against myocardial IRI in pigs by increasing enzyme activities of GR and GPx. Shen et al. (2007). demonstrated that curcumin could ameliorate warm hepatic IRI and inhibit hepatocyte apoptosis in rats through inducing the over expression of SOD and catalase. On the other hand, curcumin can increase antioxidase activities *via* altering its structures. Mofidi Najjar et al. (2017) showed that various concentrations of curcumin could increase the amount of α-helix content of catalase, which played an important role in the enhancement of the enzyme activity of catalase. Afterward, they also found that curcumin supplement resulted in the decreases in accessible surface area (ASA) and pKa of catalase, leading to the enhancement of the enzyme activity of bovine liver catalase (BLC) (Khataee et al., 2019). Therefore, curcumin can enhance the enzyme activities of various types of antioxidase, mitigating the excessive oxidative stress during reperfusion phase.

## 5 Curcumin suppresses the over-activated inflammation

Curcumin can suppress over-activated inflammation during reperfusion phase. IRI increases the percentage of inflammatory cells infiltration. Take myocardial IRI of dogs for an example, the percentage of neutrophils in endocardium increases for about 25% only after reperfusion for 5 min. Inflammatory cells such as neutrophils, monocyte-macrophages and mast cells can harm normal cells through the direct damage or secreting inflammatory mediators (Hassanzadeh et al., 2022). Taken together, curcumin can suppress inflammatory cells infiltration and decrease inflammatory mediators levels, so as to suppress the over-activated inflammation induced by IRI during reperfusion injury.

### 5.1 Curcumin suppresses inflammatory cells infiltration

#### 5.1.1 Curcumin suppresses the over expression of cell adhesion molecules

Cell adhesion molecules mainly includes integrin, selectin, ICAM-1 and vascular adhesion molecule-1 (VCAM-1), which can mediate inflammatory cells infiltration (Sawada et al., 2020). Evidences showing that curcumin can decrease the level of TNF-α-induced VCAM-1 in vascular endothelial cells and the level of ICAM-1 in aneurysmal walls (Huyut et al., 2022; Fan et al., 2019). Otherwise, (Montiel-Dávalos et al., 2017) showed that air pollutants such as inhalable particles with an aerodynamic diameter of ≤10 μm (PM10) and titanium dioxide nanoparticles (TiO2-NPs) could induce the dysfunctions and abnormal activation of endothelial cells, which led to the over expressions of ICAM-1, VCAM-1, E-selectin and P-selectin, causing the excessive inflammatory cells infiltration and damage in the endothelial cells. And their results manifested that curcumin had an anti-inflammatory role by attenuating the dysfunctions and abnormal activation of endothelial cells by the exposures to PM10 and TiO2-NPs.

#### 5.1.2 Curcumin decreases the chemokines contents

Curcumin can decrease the abnormally high contents of multiple types of chemokines induced by IRI. In IRI, the excessive inflammatory reactions are over activated, which leads to the release of a huge amount of chemokines, contributing to inflammatory cells infiltration. Jia et al., (2014) showed that curcumin treatment could lower IL-8 level in rabbit ear IRI model. (Wang et al., 2011; Kim et al., 2012) showed that curcumin decreased monocyte chemotatic protein-1 (MCP-1) level in retinal IRI and myocardial IRI respectively. In addition, (Nguyen-Ngo et al., 2020) also found that curcumin significantly suppressed TNF-induced such chemokines as C–C motif ligand 2 (CCL2), CCL3, CCL4, C-X-C motif ligand 1 (CXCL1), CXCL5 and CXCL8 expressions in human placenta, visceral adipose tissue and subcutaneous adipose tissue. Therefore, researches had confirmed both *in vitro* and vivo that curcumin could inhibit the release of a huge amount of chemokines, mitigating inflammatory cells infiltration.

#### 5.1.3 Curcumin suppresses the activation of toll-like receptors

During ischemia phase, ligands such as high mobility group box 1 (HMGB1) and RNA are significantly released by necrotic cells, which are combined by Toll-like receptors (TLRs) (Upadhyay et al., 2022). Subsequently, the excessive activation of TLRs translocates the nuclear transcription factors into nucleoli through the Myeloid Differentiation Factor 88 (MyD88) dependent signaling pathway, which produces pro-inflammatory cytokines and chemokines, inducing cascade inflammatory reactions (Zhang et al., 2021). Zou et al. (2020) showed that curcumin post-treatment could suppress TLR4 expression in the injuried lung tissues induced by limb ischaemia-reperfusion in rats. Zhang et al. (2018) also confirmed that curcumin supplementation suppressed TLR4 expression and macrophage infiltration in atherosclerosis plaque as well as protected against atherosclerosis in ApoE^−/−^ mice. Additionally, (Wang Y. et al., 2015) demonstrated that in mice sepsis models, curcumin analog L48H37 could inhibit lipopolysaccharide-induced TLR4 signaling pathway activation so that led to the decrease of downstream inflammatory mediators expression.

### 5.2 Curcumin reduces the inflammatory mediators contents

#### 5.2.1 Curcumin reduces the vasoactive amines contents

Vasoactive amines such as histamine and 5-hydroxytryptamine (5-HT) are released earliest during the inflammatory reactions, which can be suppressed by curcumin (Chong et al., 2022). Histamine mainly exists in the granules of mast cells and basophilic granulocytes, increasing vascular permeability through the combination with histamine 1 receptors in vascular endothelial cells (Matsumoto et al., 2021; Midzyanovskaya et al., 2021). *In vitro*, several researches had all confirmed that curcumin could inhibit the activation and degranulation of mast cells during reperfusion phase, which led to the suppression of histamine release, resulting in the decrease of vascular permeability (Nabil et al., 2018; Lee et al., 2018; Kong D. et al., 2020). 5-HT mainly exists in platelets, of which the release leads to the vasoconstriction (Krege et al., 2022). Arbabi Jahan et al. (2018) demonstrated that curcumin could reduce 5-HT 7 gene expression. Also, (Mohammed et al., 2020) showed that curcumin nanoparticles exerted its protective effect on the cardiotoxicity induced by doxorubicin *via* the suppression of abnormal vasoconstriction through inhibiting platelet aggregation and 5-HT release. Taken together, the protective role of curcumin in IRI partly contributes to the reductions of vasoactive amines contents and the mitigation of abnormal vascular changes.

#### 5.2.2 Curcumin reduces the contents of arachidonic acid and its metabolite

The metabolite of arachidonic acid (AA) includes prostaglandin, leukotriene (LT) and lipoxin, which are important inflammatory mediators participating in inflammation and coagulation responses (Yamaguchi et al., 2022). Dileep et al. (2011) found that curcumin and its analogs suppressed AA release *via* inhibiting the enzyme activity of phospholipase A2 (PLA2) in that PLA2 contributed to AA release by hydrolyzing membrane phospholipids. In addition, curcumin can block the synthesis of AA and its metabolite by suppressing the cyclooxygenase 2 (COX2) and lipoxygenase (LOX) (Kocaadam et al., 2017). Therefore, curcumin can alleviate IRI-induced inflammatory reactions through the reductions of AA and its metabolite.

#### 5.2.3 Curcumin reduces the pro-inflammatory factors release

TNF-α, IL-1β and IL-6 play significant roles in the initiation and promotion of inflammation, produced by activated macrophages, mast cells and endothelial cells (Oettgen, 2022; Xiao et al., 2022). When IRI occurs, as strong extracellular stimuli, ischemia, oxygen deficiency and oxidative stress can initiate the transcriptions and expressions of pro-inflammatory factors (TNF-α, IL-1β and IL-6) through the upregulation of intracellular nuclear factor κB (NF-κB) and Notch signaling pathways (Li et al., 2017; Fan et al., 2014). In turn, these types of pro-inflammatory factors also can upregulate intracellular NF-κB and Notch signaling pathways, and thus forms vicious “positive feedback” (Karatepe et al., 2009; Christopoulos et al., 2021). On one hand, (Xu et al., 2019). found that curcumin inhibited oxygen glucose deprivation/reperfusion (OGD/R)-induced cell damage by downregulating RelA p65, an important subunit of NF-κB. Also, (Liu et al., 2018) showed that curcumin exerted positive effects on hepatic IRI in mice through activating peroxisome proliferator-activated receptor γ (PPAR γ) by the downregulation of NF-κB signaling pathway. On the other hand, (Bo et al., 2020) demonstrated that curcumin post-treatment alleviated lung IRI in rats *via* the inhibition of Notch2/Hes-1 signaling pathway and the releases of pro-inflammatory factors (TNF-α and IL-1β). Taken together, curcumin treatment is related to the inhibition of pro-inflammatory releases through the regulation of inflammation-associated signaling pathways.

## 6 Curcumin inhibits the Ca^2+^ overload

Ca^2+^ overload plays an important role in the pathogenesis of IRI and it mainly occurs during reperfusion phase, of which the principal causes are the increased calcium influx, abnormal activation of protein kinase C (PKC) as well as cell membranes damage (Kashio et al., 2022; Wang et al., 2022). However, curcumin can inhibit IRI-induced Ca^2+^ overload through targeting the mechanisms mentioned above.

### 6.1 Curcumin blocks the increased Ca^2+^ influx

Increased Ca^2+^ influx greatly contributes to IRI-induced Ca^2+^ overload. During ischemia phase, intracellular anaerobic metabolism and energy deficiency both lead to the reduction of Na^+^ pump activity, resulting in the increase of intracellular Na^+^ content. During reperfusion phase, ischemic cells recover the supply of oxygen and nutritious materials and high intracellular Na^+^ content immediately activates Na^+^/Ca^2+^ exchangers, which causes the excessive Ca^2+^ influx, leading to the Ca^2+^ overload and cell injury (Junho et al., 2022). *In vitro*, (Wang Z. et al., 2020) found that curcumin protected against cerebral IRI in rats through the blockage of excessive Ca^2+^ influx by inhibiting P2X7 receptor activation, a one of the conduits for Ca^2+^ influx in dendric cells. Nazıroğlu et al. (2019) confirmed that curcumin treatment could block the increased Ca^2+^ influx in renal collecting duct cells through the downregulation of transient receptor potential M2 (TRPM2) channel which mediated oxidative stress-induced Ca^2+^ influx. Moreover, *in vivo*, (Daverey et al., 2020) also showed that curcumin mediated its protective role in spinal cord white matter hypoxia of rats through extracellular inhibition of calcium channels as well as intracellular inhibition of Ca^2+^. In turn, curcumin treatment can attenuate IRI-induced Ca^2+^ overload by blocking increased Ca^2+^ influx.

### 6.2 Curcumin suppresses PKC activation

Increased endogenous catecholamin release induced by IRI contributes to PKC activation and activated PKC promotes Na^+^/Ca^2+^ exchange and Ca^2+^ influx. Furthermore, (Chen et al., 1998) established cerebral IRI models in rats finding that the activated PKC aggravated cerebral IRI because it could lead to vasoconstriction and degenerate cytoskeleton components. Fan et al. (2019) showed that PKC β inhibitor could mitigate inflammatory cells infiltration in renal IRI tissues and promote the expression of alternatively activated macrophage (M2), a type of macrophages processing anti-inflammatory effects. To the best of our knowledge, several researches had confirmed that curcumin could suppress PKC activation. Badr et al. (2018) confirmed that curcumin analogue J1 blunted the phosphorylation of PKC-theta in the breast cancer cells. Liu et al. (1993) showed that treatment with 15 or 20 μm curcumin for 15 min could inhibit 12-0-tetradecanoyl-phorbol-13-acetate (TPA)-induced PKC activity in mice fibroblast cells. Haryuna et al. (2019) also found that intraperitoneal administration with curcumin for 3 or 8 days could reduce PKC expression in the cochlear fibroblasts of diabetic rats.

### 6.3 Curcumin maintains the integerity of cell membranes

Cell membranes are significant structures for maintaining the ionic equilibrium between the intracellular and extracellular. The destruction of cell membranes increases its permeability which causes Ca^2+^ influx following the concentrationgradient, greatly contributing to Ca^2+^ overload. Chakraborti et al. (2018) found that curcumin could alleviate ROS-induced damage on cell membranes by suppressing the phospholipase D (PLD) activity in pulmonary artery smooth muscle cells under oxidative stress condition. Other study also showed that curcumin could attenuate mitochondrial dysfunction and stabilize the cell membranes, reducing injury severity in adult models of spinal cord injury, cancer as well as cardiovascular disease (Rocha-Ferreira et al., 2019).

## 7 Curcumin affects autophagy in mitigating IRI

Autophagy-induced cell death, also termed as autosis, has been confirmed to exist in IRI and the regulation of autophagy plays an important role in the fate of cells that suffer with IRI (Shi et al., 2019; Cao et al., 2021) demonstrated that curcumin analogues, 7-(4-Hydroxy-3-methoxyphenyl)-1-phenyl-4E-hepten-3-one (AO-2) could alleviate OGD/R-induced damage on cortical neurons isolated from rats by inhibiting autophagy and cell apoptosis through an mTOR-dependent mechanism. On the contrary, (Yang et al., 2013) established the murine myocardial I/R model demonstrating that curcumin exerted its protective role against myocardial IRI through the upregulation of autophagy in murine cardiomyocytes. Additionally, *in vitro* and vivo, a series of researches had confirmed that several therapeutic agents or measure for cerebral IRI such as astragaloside IV, resveratrol and ischemic postconditioning (IPC) inhibited cerebral damage following IRI mainly through the upregulation of autophagy (Zhang et al., 2019; Sun et al., 2018; He et al., 2017). However, whether autophagy is beneficial or harmful to the IRI still remains controversial, of which the possible reasons include complicated autophagy regulation networks, differences in the durations of ischemia and reperfusion as well as different interventions, etc., (Wang X. et al., 2020; Kong D. et al., 2020). Collectively, the exact autophagy mechanisms mediating curcumin alleviating IRI still need further investigations.

## 8 Curcumin and pyroptosis in IRI

Pytoptosis, also termed as inflammation-related cell death, is an important natural immune response of the body (Hu et al., 2022; Naryzhnaya et al., 2022). Although there are no researches reporting the possible relationships between pyroptosis and curcumin alleviating IRI yet, the inhibition of pyroptosis has been confirmed to mitigate IRI. Nucleotide-binding oligomerization domain-like receptor protein 3 (NLRP3) is the key protein of IRI-induced pyroptosis (Luan et al., 2022). In the development and progression of IRI, increased NLRP3 level induces violent pytoptosis, which is led by the upregulation of NF-κB signaling pathway, increased mitochondrial fragments, impaired autophagy functions and high ROS levels, etc., (Song et al., 2018; Yu et al., 2022). Therefore, a series of researches had confirmed that several types of TCM such as total glucosides of paeonia (TGP), emodin, β-asarone and gastrodin could alleviate myocardial IRI and intestinal IRI *via* inhibiting pyroptosis in cardiocytes and intestinal mucosal epithelial cells (Zheng et al., 2019; Ye et al., 2019; Xiao et al., 2020; Sun et al., 2019). As for curcumin treatment, (Yin et al. 2020) found that curcumin could attenuate necrotising microscopic colitis by inhibiting pyroptosis in newborn rats. Yu et al. (2020) found that curcumin could protect against doxorubicin-induced cardiac injury *via* suppressing pyroptosis in mice. Taken together, the inhibition of pyroptosis by curcumin might be one of the most significant mechanisms of allevating IRI and more high-qualified researches should be performed.

## 9 Curcumin and ferroptosis in IRI

Ferroptosis, a new type of regulated cell death that has been discovered recently, which is induced by iron-dependent lipid peroxidation (Li H. et al., 2020; Ma et al., 2023). Currently, it has been widely accepted that it plays an importantly detrimental role in many IRI models (Yan, 2020). As mentioned above, IRI is accompanied by oxidative damage and the accumulation of ROS. Additionally, Zhao et al., (2018) and Scindia et al., (2019) both had found that IRI could cause elevated intracellular iron levels, which was induced by excessive oxidative stress, and iron overload was also a main source of oxidative stress in turn, aggravating IRI. These pathomechanisms related to IRI are main causes of ferroptosis. Elevated intracellular iron promoted lipid oxidation by Fenton reaction, contributing to the induction of ferroptosis (Hirschhorn et al., 2019). Therefore, given the relationships between ferroptosis and IRI, a series of researches had demonstrated the protective role of iron chelators against IRI models. Tuo et al. (2017) showed that tau alleviated cerebral IRI in rats by ferroptotic inhibition, of which the main mechanism was mediating stroke-induced iron accumulation and outcome. *In vivo*, (Fang et al., 2019) also showed that ferrostatin-1, a classical type of iron chelators, could ameliorated heart failure induced by both acute and chronic myocardial IRI in mice. In addition, (Linkermann, 2016) concluded that such iron chelators as desferoxamine, ferrostatin-1, liproxstatin and the compound 16–86 could greatly attenuate tubular cell necrosis and synchronized death of renal tubules caused by renal IRI. *In vitro*, (Li et al., 2018). demonstrated that supplement with liproxstatin-1 and deferoxamine could block testicular IRI-induced cell death of germ cells and Sertoli cells. To our best understanding, curcumin treatment has been reported to inhibit ferroptosis. Guerrero et al. (2019) had showed that curcumin treatment could mitigate renal damage relating to rhabdomyolysis through inhibting ferroptosis-induced cell death. Li R et al. also showed that curcumin could suppress the growth of breast cancer cells and decrease the survival rate of osteosarcoma cells by inducing ferroptosis-mediated cell death. Collectively, curcumin can be both ferroptosis inhibitor and ferroptosis trigger (Tang et al., 2021). However, whether curcumin can exert its protective role against IRI by ferroptosis-associated mechanisms should be explored further.

## 10 The side effect of curcumin usage and the suggested administrations

There are also some side effects of curcumin. For example, external application of curcumin might cause contact dermatitis and urticaria (Chen L. et al., 2022). And its possible explantation is that external application of curcumin can induce the allergic reaction of skin. Oral administration of curcumin might cause gastrointestinal pain, nausea, vomit and liver poisoning (Xie et al., 2022). Otherwise, the overdose of curcumin can aggravate the cholecystolithiasis in that curcumin is mainly metabolized by the liver, so that the excessive administration of curcumin can induce liver dysfunction. In addition, curcumin can inhibit the coagulation process so that people suffered from hemorrhagic diseases, postoperative patients and pregnant women should take curcumin with great caution (Chen et al., 2022). Futhermore, curcumin can reduce the blood pressure and blood glucose so that people who take hypotensor and hypoglycemic drugs are forbidden to take curcumin. In clinics, take as mycardial infarction patients an example. In my opinion, administration of curcumin should act as an auxiliary role in treating mycardial infarction and should be given right after the ischemic event (Li J. et al., 2020). Taken together, there is a long way from scientific to clinical.

## 11 The limitations of the review

Our review also had a few limitations. For instance, the study did not review the molecular structure of curcumin. Besides, this review did not include our own exprimental data. In addition, the characteristics of the molecular structure of curcumin and relevant experiments should be investigated in our next aticle in the future.

## Conclusion

In conclusion, curcumin has been confirmed to alleviate IRI. During ischemia phase, curcumin can suppress the excessive upregulation of HIF-1α and anaerobic metabolism, reducing ischemia-induced injury on cells. During reperfusion phase, curcumin can inhibit the excessive oxidative stress through decreasing free radicals levels and increasing antioxidase activities. Simultaneously, curcumin can mitigate the excessive inflammation through suppressing inflammatory cells infiltration and decreasing inflammatory mediators contents. Additionally, curcumin also can inhibit intracellular Ca^2+^ overload by blocking the increased Ca^2+^ influx, suppressing PKC activation and maintaining the integerity of cell membranes.
